# Classification of offshore wind grid-connected power quality disturbances based on fast S-transform and CPO-optimized convolutional neural network

**DOI:** 10.1371/journal.pone.0314720

**Published:** 2024-12-05

**Authors:** Minan Tang, Hongjie Wang, Jiandong Qiu, Zhanglong Tao, Tong Yang

**Affiliations:** 1 College of New Energy and Power Engineering, Lanzhou Jiaotong University, Lanzhou, China; 2 College of Electrical and Mechanical Engineering, Lanzhou Jiaotong University, Lanzhou, China; 3 College of Automation and Electrical Engineering, Lanzhou Jiaotong University, Lanzhou, China; Xi’an University of Technology, CHINA

## Abstract

The large-scale integration of offshore wind power into the power grid has brought serious challenges to the power system power quality. Aiming at the problem of power quality disturbance detection and classification, this paper proposes a novel algorithm based on fast S-transform and crested porcupine optimizer (CPO) optimized CNN. Firstly, the intrinsic mechanism and waveform characteristics of offshore wind power grid-connected disturbances are analyzed, and the simulated disturbance signals are feature extracted and time-frequency diagrams are obtained by fast S-transform. Secondly, the CPO algorithm is used to optimize the convolutional neural network and determine the best hyperparameters so that the classifier achieves the optimal classification performance. Then, the CPO-CNN classification model is used for feature extraction and feature selection of the time-frequency diagrams and classification of multiple power quality disturbances. Finally, a simulation experimental platform is established based on MATLAB to perform simulation verification and comparative analysis of power quality disturbance classification. The experimental results show that the model established in this paper is effective, and the classification accuracy is improved by 3.47% compared with the CNN method, which can accurately identify the power quality disturbance signals, and then help to assess and control the power quality problems.

## Introduction

**Fig 1:**
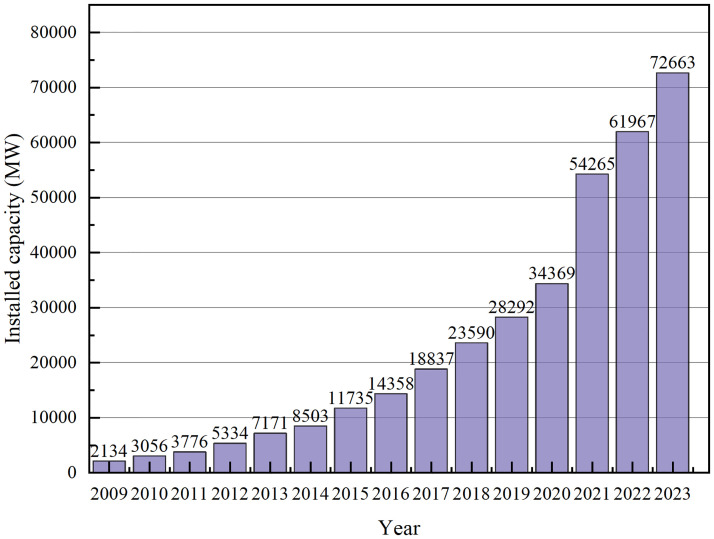
Trends in installed offshore wind power capacity.

In recent years, the proportion of multiple new energy generation modes in the grid has gradually increased [1], with offshore wind power becoming one of the most popular renewable energy sources due to its abundant and stable wind resources. By the end of 2023, the global installed capacity of offshore wind power has reached an impressive 72 gigawatts (GW), and it is expected to grow significantly, reaching 233 GW by 2030 [2], with the change in installed capacity shown in Fig 1. Due to the intermittent nature of wind energy, the variable operating conditions of offshore wind turbines, the high penetration of renewable energy in the grid, and the use of power electronic equipment, the continuous growth of installed capacity leads to the increasingly prominent problem of grid-connected power quality disturbances of offshore wind power, which seriously affects the stable operation of the power system, and even results in serious safety accidents and economic losses. Power quality (PQ) has also become a primary concern. Therefore, effective and accurate identification and classification of offshore wind power grid-connected PQ disturbances is of great significance for grid fault management and power quality improvement.

Typical PQ problems when operating offshore wind energy systems include voltage transients, voltage flicker, harmonics, voltage sags, voltage swells and voltage interruptions [3]. Existing PQ disturbances not only appear in the form of a single disturbance, but also often appear as two or even three composite disturbances. Therefore, the study of compound PQ disturbance events is particularly important for power quality management, and more helpful for the next step of power quality optimization [4].

Power quality disturbance signal recognition includes three processes: feature extraction, feature selection and classification. Currently, scholars around the world have taken two main approaches to the study of power quality disturbances (PQDs): a two-step approach based on signal analysis algorithms and classifiers, and a deep learning approach based on artificial intelligence [5]. The two-step approach is the most widely studied, which first resolves important time-frequency features by signal analysis algorithms such as short-time Fourier transform (STFT) [6], wavelet transform (WT), S-transform [7], Hilbert Huang transform (HHT) and empirical modal decomposition [8] as well as their corresponding improved algorithms, then determines the set of eigenvalues by using artificial experience and learns the mapping relationship between sequences and labels by means of a classifier, and finally realizes classification. Some of the commonly used classifiers are decision trees [9], support vector machines [10] and BP neural networks [11]. Among these traditional signal processing methods, STFT is not suitable for analyzing transient signals due to spectral aliasing and leakage phenomena. WT is highly susceptible to noise interference, so it is difficult to distinguish between signals such as temporary voltage rises and drops. HHT is suitable for analyzing mutant signals but the overall performance is not good. The S-transform combines the advantages of WT and STFT by replacing the wavelet basis function with a Gaussian window to solve the limitation of fixed window widths in STFT and also has the advantage of a fixed window width as well as better noise immunity, but the time complexity and space complexity are high, which affects the operation speed [12–14]. The improved fast S-transform solves this problem well, possesses the advantages of S-transform and improves the operation speed at the same time, and shows excellent performance in signal processing. Literature [15] used a combination of S-transform and probabilistic neural network (PNN) to classify 11 types of disturbances. Literature [16] proposed an algorithm combining S-transform and Artificial Neural Network (ANN) and decision tree for feature extraction and disturbance classification. Literature [17] used fast S-transform to extract features and an automatic decision tree (DT) construction algorithm to select the optimal set of features for identifying PQ disturbances. Literature [18] combined WT with Support Vector Machine (SVM) to obtain six levels of decomposition coefficients using WT, eight statistical methods to extract features of each disturbance signal, and also used SVM to design a classifier. Although these traditional methods achieve good classification results, they seriously require manual experience to adjust the feature dimensions and the inputs required by the classifiers, which restricts the optimal feature extraction of the disturbances, and the classification is limited to a limited number of categories, which is a limitation. It generally performs relatively poorly in dealing with multiple disturbance composites. With the complexity of the disturbances, the traditional methods are no longer able to meet the grid’s classification needs for PQDs. The use of deep neural networks to directly perform feature extraction and feature selection on the time-frequency features of signal processing before classification has become an important method [19]. Deep learning has excellent self-training and feature extraction capabilities, and the deep features it describes can adequately identify the disturbance categories. The method is mainly represented by neural networks, firstly, the original PQDs one-dimensional signals are transformed into two-dimensional images by upscaling algorithms, and then input into networks such as CNN to extract deep features and classify them [20]. Some other scholars have optimized the parameters of the classification model using swarm intelligence algorithms such as particle swarm algorithm (PSO) [21, 22], genetic algorithm (GA) [23], and whale algorithm (WOA) [24] to ensure better classification performance, but there is still room for improvement in its classification accuracy. Literature [25] uses variational modal decomposition (VMD) to pre-process the interference for denoising, and then uses the improved gray wolf optimization algorithm (IGWO) to optimize the support vector machine (SVM) model parameters, which ultimately achieves a higher classification accuracy. Literature [26] takes PV microgrid as the background, obtains the time-frequency analysis results through S-transform, and then optimizes the BP neural network using SABO algorithm, and finally performs automatic classification and identification. However, the BP neural network feature extraction and parameter processing capabilities are limited, especially underperforming in high-dimensional data and complex tasks. Literature [27], on the other hand, uses the PSO algorithm to optimize the CNN parameters so that the model achieves higher classification accuracy. Literature [28] proposed a method to automatically identify six PQDs by CNN through continuous wavelet transform (CWT) and CNN for the power quality problem of wind and photovoltaic power generation systems. However, it does not use an optimization algorithm to optimize the model parameters. Among many classification methods, CNN is widely regarded as the most popular PQ event classification method, which uses neural network convolution with lattice-like topology to analyze the PQDs signals.

There have been many papers on PQDs, with a large proportion of traditional power systems as the background, and the impact of offshore wind power grid integration on power quality issues is beginning to be mentioned and researched. In view of the great progress of offshore wind power and the rapid innovation of algorithms and detection techniques, the detection and classification of PQDs have been in need of more in-depth research and discussion. Currently, there are fewer studies on offshore wind grid-connected PQDs signals, and the detection and classification identification accuracy are low. In order to solve this problem, this paper proposes a new method for offshore wind power grid-connected PQDs classification. The novelty of this study lies in the current research status of power quality disturbances in the context of offshore wind power grid connection, emphasizing the impact of offshore wind power on power quality, and innovatively proposing a combination of fast S-transform, convolutional neural network, and crested Porcupine Optimizer to establish a new classification and prediction model for solving the problem of detecting and classifying multiple and complex power quality disturbances that are short in time and harmful in nature. The method mainly focuses on the problem of accurate detection and identification of complex disturbances. Aiming at the difficulties of high complexity of time-frequency analysis methods such as S-transform, difficulty in determining the parameters of CNN network, and low classification accuracy of traditional neural network model, the fast S-transform is used to reduce the time and space complexity of signal processing and improve the real-time identification capability. At the same time, CPO optimization algorithm is used to optimize the CNN in an attempt to overcome the deficiencies of the algorithm’s searching ability and classifier performance, so as to establish the CPO-CNN neural network classification model, which effectively exerts the advantages of low complexity, fast computation speed, and good optimization effect, and achieves the effect of fast detection speed and high classification accuracy. The experimental results show that the classification accuracy of the proposed method is significantly improved, and it is an effective classification method, which provides a new way of thinking for the classification of offshore wind power grid-connected power quality disturbances.

The innovations are reflected in the following:

1. This paper analyzes the impact of offshore wind energy on the types of PQDs by taking offshore wind power grid connection as the research background. Meanwhile, the problem of PQDs detection and categorization in this context is studied in depth. It provides prerequisites for subsequent offshore wind power quality control strategies and improvement methods.
2. The traditional S-transform signal processing algorithm has high time complexity and space complexity, which makes it difficult to achieve real-time and efficient power quality disturbance identification. To address this problem, this paper utilizes the fast S-transform (FST) method, which improves the computing speed by inverting the fast Fourier transform for the main frequency points and the disturbance frequency points.
3. In this paper, a novel algorithm combining CPO optimization algorithm and convolutional neural network is proposed for classification prediction of PQDs types. This classification prediction algorithm can effectively improve the generalization ability and classification accuracy of the model.

**Fig 2:**
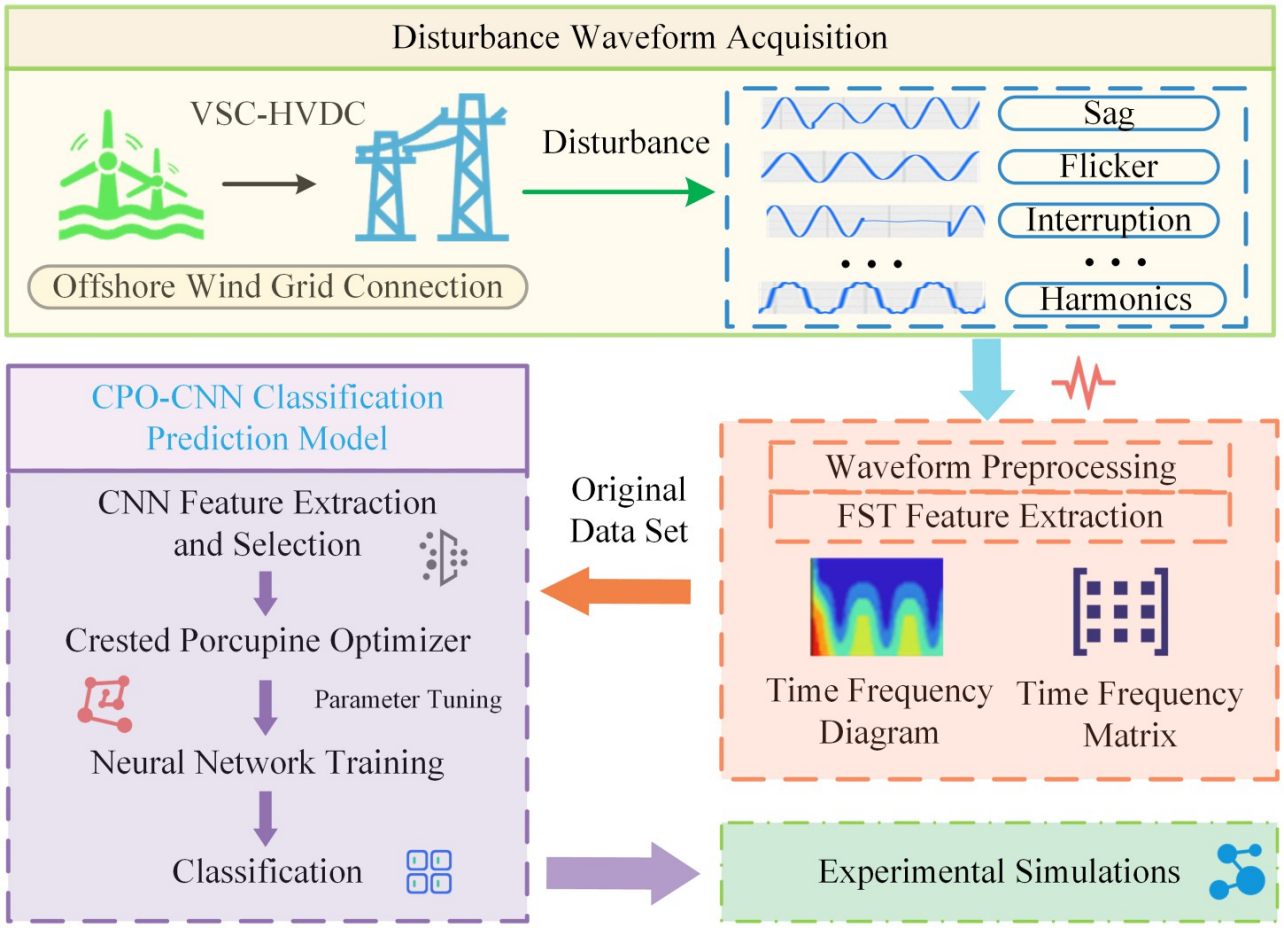
The structural layout sketch of this paper.

The structure of this paper is shown as follows: The Offshore wind power grid-connected PQDs problem section specifically introduces the main PQDs problems generated by offshore wind power grid integration, describes in detail the structure of offshore wind power grid integration as well as the impact on the power quality of the grid, and finally describes the disturbance mathematical model as well as the waveform features. The Fast S-transform time-frequency feature extraction model section generates a two-dimensional time-frequency image by establishing a fast S-transform (FST) feature extraction model for signal processing of one-dimensional disturbance waveforms, and a large number of disturbance features exist in the time-frequency matrix. The CPO-CNN classification prediction model section establishes a CPO-CNN classification prediction model to optimize the convolutional neural network hyperparameters using the CPO optimization algorithm for better feature extraction, feature selection and classification identification. The Experimental simulation and analysis section conducts experimental simulation and analysis, and compares and analyzes with other optimization algorithms while simulating to ensure the superiority of the classification performance of the model proposed in this paper. The Conclusion section concludes the paper. The structural layout sketch of this paper is shown in Fig 2.

## Offshore wind power grid-connected PQDs problem

Power quality disturbances mainly include steady state disturbances and transient disturbances. When offshore wind power is connected to the grid, due to its special operating environment and technical characteristics, it often causes power quality disturbance problems, of which transient PQDs problems are more prominent, and is also the focus and difficulty of power quality analysis. The generation of complex disturbances has an adverse effect on the power quality, reliability and economic operation of the grid, and these problems are particularly prominent in the context of offshore wind power grid integration.

### Impact of offshore wind power grid integration on grid power quality

In power grids integrating offshore wind energy, the variable behavior of disturbance sources such as environmental factors and loads makes disturbance analysis challenging [2]. First, the energy output of offshore wind farms tends to be highly variable due to harsh weather conditions, thus creating unpredictable power supply levels in power system operations. Secondly, wind farms are subject to electromagnetic interference (EMI) from different sources such as nearby electrical equipment, communication systems and radio frequency equipment, all of which can cause EMI. Thirdly, the inherent nonlinear properties of electromagnetic equipment and components in offshore wind farms can cause harmonic pollution. Finally, electromagnetic interactions between WTGs and the grid may result in resonance phenomena, which in severe cases can lead to system failures and blackouts [29]. Therefore, it becomes crucial to identify the types of PQDs involved.

The output power of wind power fluctuates with changes in wind speed, resulting in unstable grid voltage. Coupled with the rapid voltage changes caused by the frequent start and stop of high-power equipment in wind power plants, these factors can produce voltage fluctuations and voltage flicker, which in turn lead to unstable operation and shortened life of electrical equipment. The nonlinear loads introduced by power converters and transformers, electronic components, etc. in the offshore wind power system will generate a large number of harmonics, leading to overheating, increased noise, reduced efficiency and even damage to equipment [2]. When a short circuit or disconnection occurs at the wind farm grid connection point, it will cause transient voltage disturbances such as voltage sags and voltage swells, which will affect the normal operation of the equipment. In addition to the above power quality problems, there are also voltage three-phase imbalance, frequency deviation, voltage deviation and so on. The existence of these problems also affects the safe operation of offshore wind farms.

### Offshore wind grid integration structure

**Fig 3:**
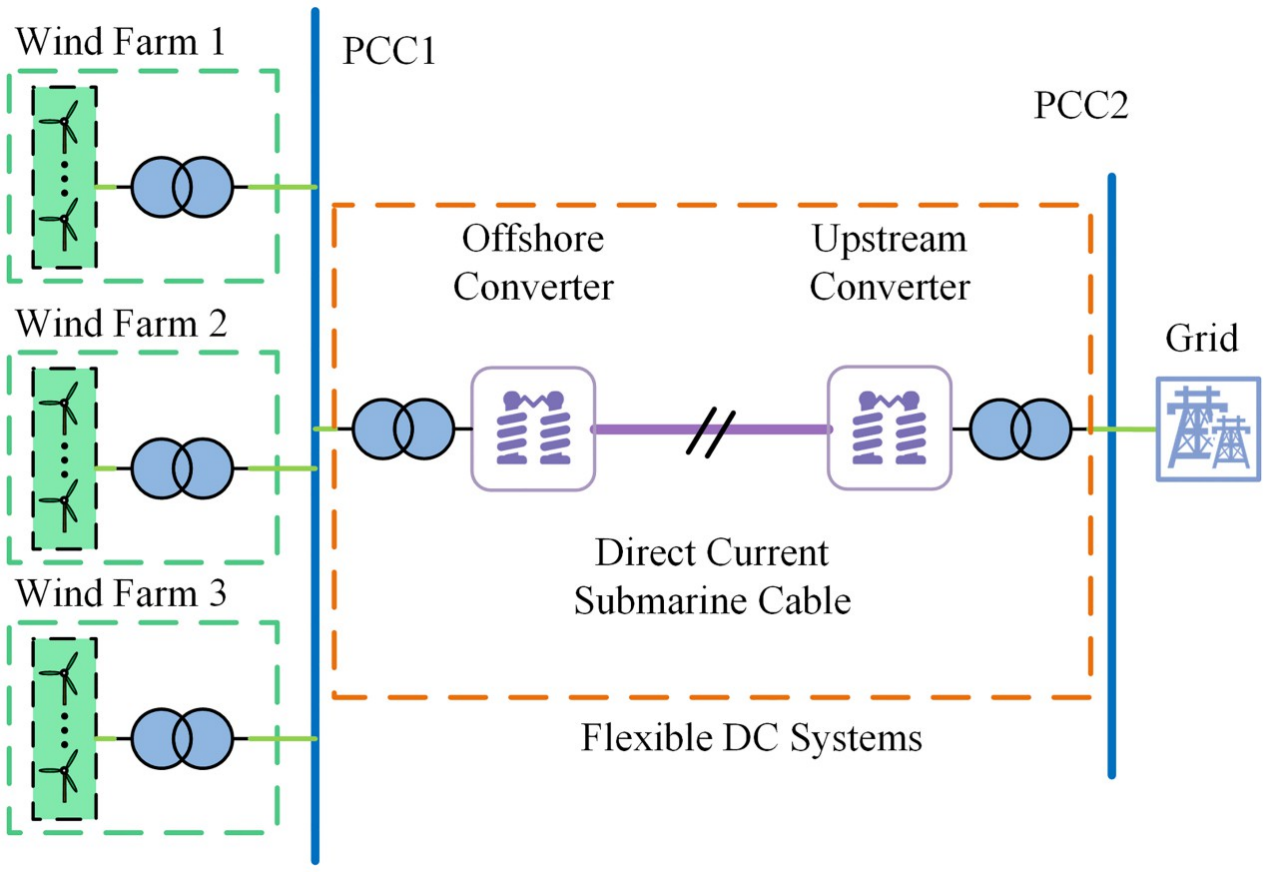
Offshore wind power plant via flexible straight grid connection structure diagram.

In recent years, flexible DC transmission technology has been developed rapidly. The offshore wind power grid-connected system based on VSC-HVDC has been widely applied with many advantages such as flexible control mode, no phase change failure problem, and strong fault ride-through capability [30, 31]. The offshore wind power via flexible direct grid-connected system mainly includes three parts: offshore wind farm, flexible DC system and AC grid, and the flexible DC system includes offshore converter station, DC cable and onshore converter station. The structure diagram of offshore wind power plant via flexible direct grid connection is shown in Fig 3. The offshore wind power plant generates electricity first, and the electricity from multiple wind farms is gathered at the public coupling point, converted to DC by the offshore converter station, and then delivered to the power through the submarine cable, and converted to AC by the onshore converter station, and finally connected to the power grid.

### Mathematical modeling of PQDs

Power quality disturbance signals are difficult to describe in words, and mathematical modeling can accurately generate single or compound power quality disturbance waveforms in simulation software. According to the international standard IEEE 1159-2019, common single disturbances are generally categorized into eight types, which are voltage swells, voltage sags, voltage interruptions, transient pulses, transient oscillations, voltage gaps, flicker and harmonics [32–34]. Multiplying or adding these single signals can generate a variety of dual or even triple power quality disturbance signals. These signals are the result of a single disturbance acting together. Common mathematical models of single power quality disturbance signals are shown in Table 1.

**Table 1:** Mathematical model of power quality disturbance signal.

Disturbances	Mathematical Model	Parametric
Normal	*h*(*t*) = *A* sin (*ωt*)	*A* = 1 (*pu*), *f* = 50*HZ*
Sag	*h*(*t*) = *A*[1 − *α*(*u*(*t* − *t*_1_) − *u*(*t* − *t*_2_))] sin (*ωt*)	0.1 ≤ *α* ≤ 0.9, *T* ≤ *t*_2_ − *t*_1_ ≤ 9*T*
Swell	*h*(*t*) = *A*[1 + *α*(*u*(*t* − *t*_1_) − *u*(*t* − *t*_2_))] sin (*ωt*)	0.1 ≤ *α* ≤ 0.8, *T* ≤ *t*_2_ − *t*_1_ ≤ 9*T*
Interruption	*h*(*t*) = *A*[1 − *α*(*u*(*t* − *t*_1_) − *u*(*t* − *t*_2_))] sin (*ωt*)	0.9 ≤ *α* ≤ 1, *T* ≤ *t*_2_ − *t*_1_ ≤ 9*T*
Flicker	*h*(*t*) = *A*[1 + *α*_*f*_ sin (*βωt*)] sin (*ωt*)	0.1 ≤ *α*_*f*_ ≤ 0.2, 5 ≤ *β* ≤ 20*Hz*
Harmonics	*h*(*t*) = *A*[*α*_1_ sin (*ωt*) + *α*_3_ sin (3*ωt*) + *α*_5_ sin (5*ωt*) + *α*_7_ sin (7*ωt*)]	0.05 ≤ *α*_3_, *α*_5_, *α*_7_ ≤ 0.15, ∑(*α*_*i*_)^2^ = 1
Impulsive transient	$h(t)=A[\text{sin}\;(\omega t)+\alpha \;\text{exp}\;(\frac{t-t_{1}}{\tau })(u(t-t_{1})-u(t-t_{2}))]$	0.1 ≤ *α* ≤ 0.8, *T*/20 ≤ *t*_2_ − *t*_1_ ≤ *T*/10, 8 ≤ *τ* ≤ 40
Oscillatory transient	$h(t)=A[\text{sin}\;(\omega t)+\alpha \;\text{exp}\;(-\frac{t-t_{1}}{\tau })\;\text{sin}\;\omega_{n}(t-t_{1})(u(t-t_{1})-u(t-t_{2}))]$	0.1 ≤ *α* ≤ 0.8, 0.5*T* ≤ *t*_2_ − *t*_1_ ≤ 3*T*, 8 ≤ *τ* ≤ 40, 300 ≤ *f*_*n*_ ≤ 900*Hz*

**Fig 4:**
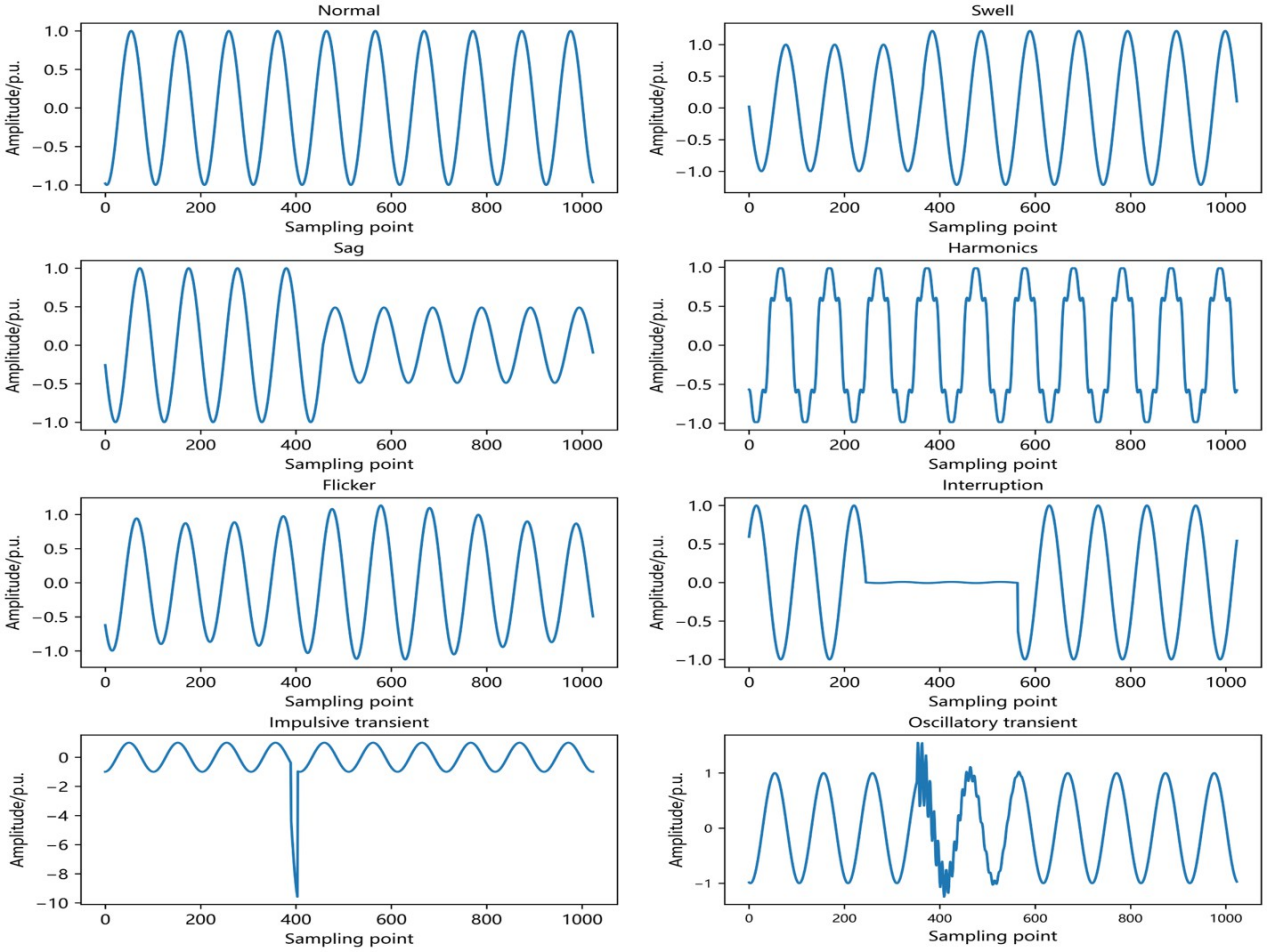
Common single power quality disturbance waveforms.

The normal signal and common single power quality disturbance waveforms are shown in Fig 4.

The composite power quality disturbance signal can be simulated and generated by Eq (1).

$$\begin{array}{c} \begin{array}{cc} x(t) & =[sag(t),swl(t),itr(t)]\times [flk(t)]\times \\ & \left\{\text{sin}\;(t)+[hmy(t)]+[nth(t)]+[noise(t)]\right\}\\ & +[osc(t),imp(t)] \end{array} \end{array}$$ (1)

Where sin(*t*) is the standard sinusoidal function, *sag*(*t*), *swl*(*t*), *itr*(*t*) is the voltage swell, voltage sag, voltage interruption disturbance factors, *imp*(*t*), *osc*(*t*), *nth*(*t*) is the transient pulse, transient oscillation, voltage gap and other disturbance factors, *flk*(*t*), *hmy*(*t*) is the flicker and harmonic disturbance factors, *noise*(*t*) is the noise signal [4].

## Fast S-transform time-frequency feature extraction model

Single and complex power quality disturbance signals appear in offshore wind grid-connected systems from time to time. When the disturbance signals are obtained from the grid, how to preprocess the original signals becomes the primary problem of identification and classification. In this paper, the fast S-transform is used to extract time-frequency features from the original signal, decompose the one-dimensional signal waveform into a two-dimensional time-frequency map, and generate the time-frequency matrix containing the feature information. The fast S-transform feature extraction model is established by improving the S-transform.

### S transform

Time-frequency feature extraction using signal processing algorithms on the original signals is the first step in disturbance classification. For a series of unsteady signals, the S-transform can accurately describe them in both time and frequency domains [35, 36]. The S-transform combines the advantages of the wavelet transform and the Fourier transform, and is a reversible time-frequency analysis method. The basic idea is to replace the wavelet basis function with a Gaussian window, which improves the defect of fixed window width, has superior time-frequency characteristics and is not easily affected by noise [37].

Let the disturbance signal be *h*(*t*), and the S-transformed expression be:

$$\begin{array}{c} S(\tau ,f)=\int_{-\infty }^{\infty }h(t)\omega (\tau -t,\sigma )e^{-i2\pi ft}dt \end{array}$$ (2)

Where t and f are the time and frequency in the S-domain, respectively, *h*(*t*) is the signal to be analyzed, *ω*(*τ* − *t*, *σ*) is the Gaussian window function, *τ* is the time translation factor, *σ* is the window function scale factor, which is the bridge between the window width and frequency. By adjusting the scale factor, the window width can be improved to achieve superior time-frequency characteristics [39].

The Gaussian window function expression is:

$$\begin{array}{c} \omega (\tau -t,\sigma )=\frac{1}{\sigma \sqrt{2\pi }}e\frac{-{\left(\tau -t\right)}^{2}}{2\sigma^{2}} \end{array}$$ (3)

Window function scale factor:

$$\begin{array}{c} \sigma =\frac{1}{\left|f\right|} \end{array}$$ (4)

The discrete expression of the S-transform is given by (*f* → *n*/*NT*, *τ* → *jT*):

$$\begin{array}{c} \left\{ \begin{array}{cc} S(jT,\frac{n}{NT})=\sum_{m=0}^{N-1}H(\frac{m+n}{NT})G(m,n)e^{i2\pi mj/N}, & n\neq 0\\ S(jT,0)=\frac{1}{N}\sum_{m=0}^{N-1}H(\frac{m}{NT}), & n=0 \end{array}\right. \end{array}$$ (5)

Where $G(m,n)=e^{-2\pi^{2}m^{2}\sigma^{2}f^{2}/n^{2}}$, N is the number of sample sampling points, T is the sampling time interval, *j* = 0, 1, ⋯, N, *G*(*m*, *n*) obtained from the Gaussian window function after discrete fast Fourier transform, $H(\frac{m+n}{NT})$ obtained from the input signal after discrete fast Fourier transform displacement.

Analyzed from the perspective of feature extraction of perturbed signals, the feature extraction of disturbance only needs to extract features for a specific frequency or frequency range, due to the large amount of redundancy in the computational process of ST in dealing with perturbed signals with a sampling point number of N. Its time complexity is *O*(*N*^3^), and its space complexity is *O*(*N*^2^), which is not good for small images and longer one-dimensional perturbed signals to achieve. Fast S-transform can well solve the large amount of redundant computation and make the computation faster [38–40].

### Fast S-transform

A new *α* domain is introduced on the basis of the S-transform, which is obtained from the generalized S-domain Fourier transform.

$$\begin{array}{c} \alpha (f^{\prime },f)=FT(S(\tau ,f))=\int_{-\infty }^{\infty }S(\tau ,f)e^{-i2\pi f^{\prime }\tau }d\tau \end{array}$$ (6)

Where *f*′ is the pair of transformed variables for *τ* in the S-domain. The defining equation of the S-transformation can be obtained by bringing into Eq (7):

$$\begin{array}{c} \alpha (f^{\prime },f)=G(f^{\prime }+f)\cdot W(f^{\prime },\sigma ) \end{array}$$ (7)

Where *G*(*v*′) and *W*(*f*′, *σ*) are the Fourier transforms of the original signal and the window function with respect to *τ*. Thus the S-domain signal is:

$$\begin{array}{c} S(\tau ,f)=\int_{-\infty }^{\infty }\alpha (f^{\prime },f)e^{j2\pi f^{\prime }\tau }df^{\prime } \end{array}$$ (8)

The essence is actually the Fourier inverse transform of *α*. The FFT of the original signal is cyclically shifted and multiplied by the frequency domain window function to obtain the ST of the original signal. By applying the FST algorithm to the time-domain signal sequence, the time complexity is reduced to *O*(*N* log *N*) and the space complexity is reduced to *O*(*N*). This algorithm is much faster in computation compared to the traditional S-transform [41].

### Model building

The power quality disturbance signals generated by offshore wind power grid integration are essentially a series of unsteady signals, which are mainly transient disturbances, occurring in a short period of time and at a fast speed, and the spectral information of the disturbance signals in different time periods can be accurately analyzed by using the fast S-transform. The spectrogram and time-frequency matrix contain a lot of feature information, through the fusion and selection of these features, it is possible to classify the complex disturbance signals, and then detect the power quality problems in the power grid.

Firstly, the power quality waveform signals in the process of offshore wind power grid-connection are firstly acquired by the grid monitoring equipment, and the collected raw data are preprocessed to ensure the consistency of data quality. Secondly, a fast Fourier transform is performed on the preprocessed signal. Then a Gaussian window function is constructed in the frequency domain, and the spectrum of the signal is weighted with the Gaussian window function. The Fourier inverse transform is performed on the weighted signal to obtain the signal in the time domain, and at the same time, the time-frequency matrix is generated, and the time-frequency features are extracted in the time-frequency matrix. Finally the time-frequency plot is generated to reflect the amplitude distribution of the signal at different times and frequencies.

**Fig 5:**
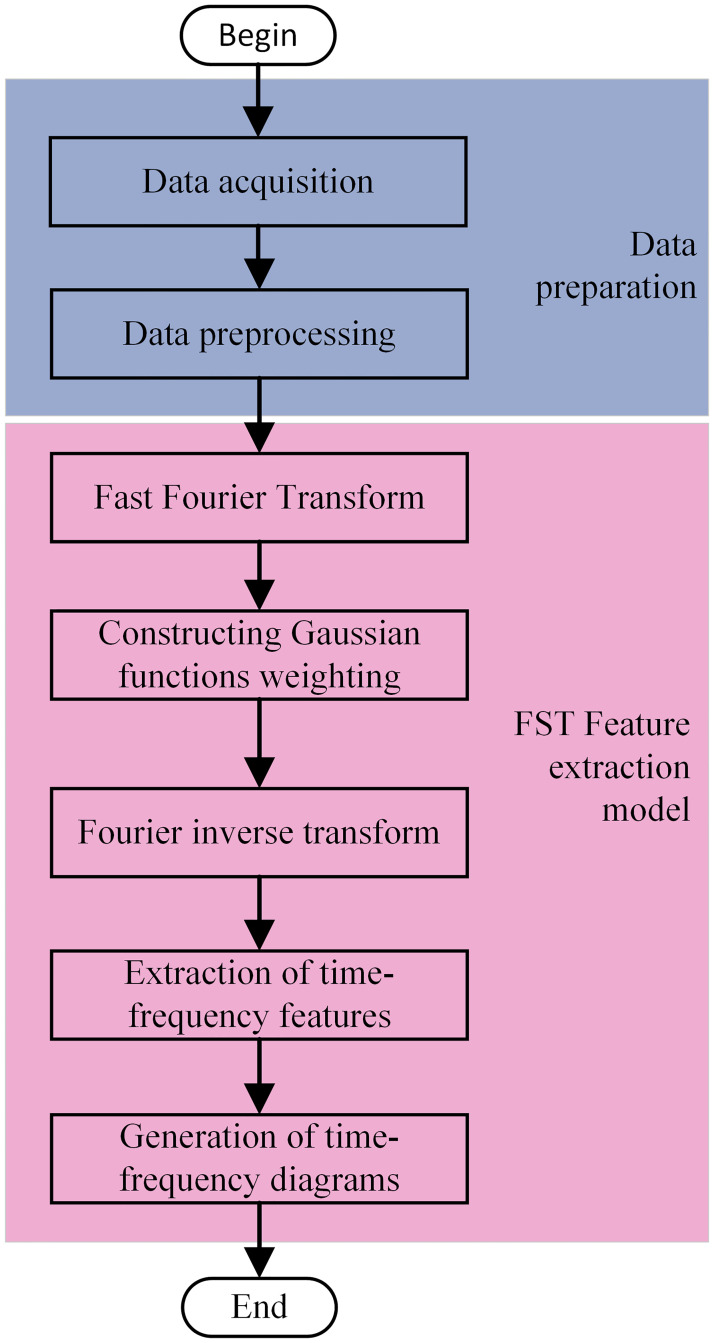
Time-frequency feature extraction flowchart.

The flowchart of time-frequency feature extraction of offshore wind grid-connected power quality disturbance signals is shown in Fig 5.

## CPO-CNN classification prediction model

The original signal will be decomposed into a series of 2D time-frequency images after FST, and the CPO-CNN classification prediction model is established to further process them. The time-frequency image is used as the input of the model, and the classification prediction result is used as the output of the model, which finally realizes accurate and effective power quality disturbance identification and classification.

### Convolutional neural network

**Fig 6:**
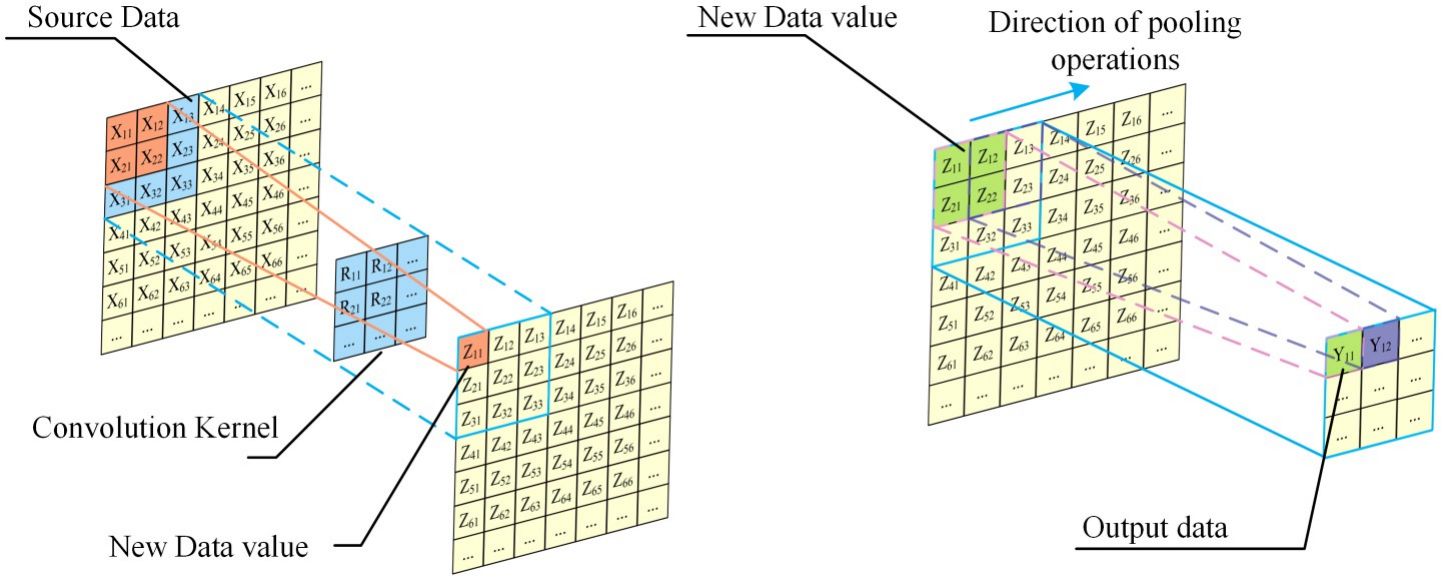
Schematic diagram of CNN convolutional and pooling layer.

In this paper, CNN is utilized to identify the information contained in the learned time-frequency map and time-frequency matrix to capture the correlation between the input features and power quality disturbances. Convolutional neural network is a multilayer supervised learning neural network that contains convolutional computation and has a deep structure, which consists of three parts: input layer, hidden layer, and output layer [42–45]. The hidden layer generally contains multiple convolutional blocks, and the convolutional blocks generally include a convolutional layer, a pooling layer, an activation function, and a fully connected layer [46]. Among them, the convolutional layer and pooling layer are the core modules to realize feature extraction, and the pooling layer and fully connected layer are the key to realize feature selection. Finally, the multiclassification task is performed by the output layer using softmax function. The advantage is that the local links and weights are shared, which can reduce the complexity of the model [47, 48]. The schematic diagram of CNN convolutional and pooling layers is shown in Fig 6.

### Crested Porcupine Optimizer

The Crested Porcupine Optimization (CPO) algorithm is a novel meta-heuristic algorithm inspired by the multiple defensive behaviors of the crowned porcupine, proposed by Abdel-Basset et al. Unlike other optimization algorithms inspired by predator attack behaviors, CPO draws inspiration from modeling the defensive behaviors adopted by prey for survival. When the crowned porcupine is threatened by a predator, it generates four different protective mechanisms from least aggressive to most aggressive, namely visual, sound, odor, and physical attacks [49]. The algorithm makes full use of these four defensive behaviors of the crown porcupine, while integrating a cyclic population reduction technique to simulate the activation of the defense mechanisms when the crested porcupine is threatened, which in turn improves the speed of convergence of the optimal solution and the population diversity.

**Fig 7:**
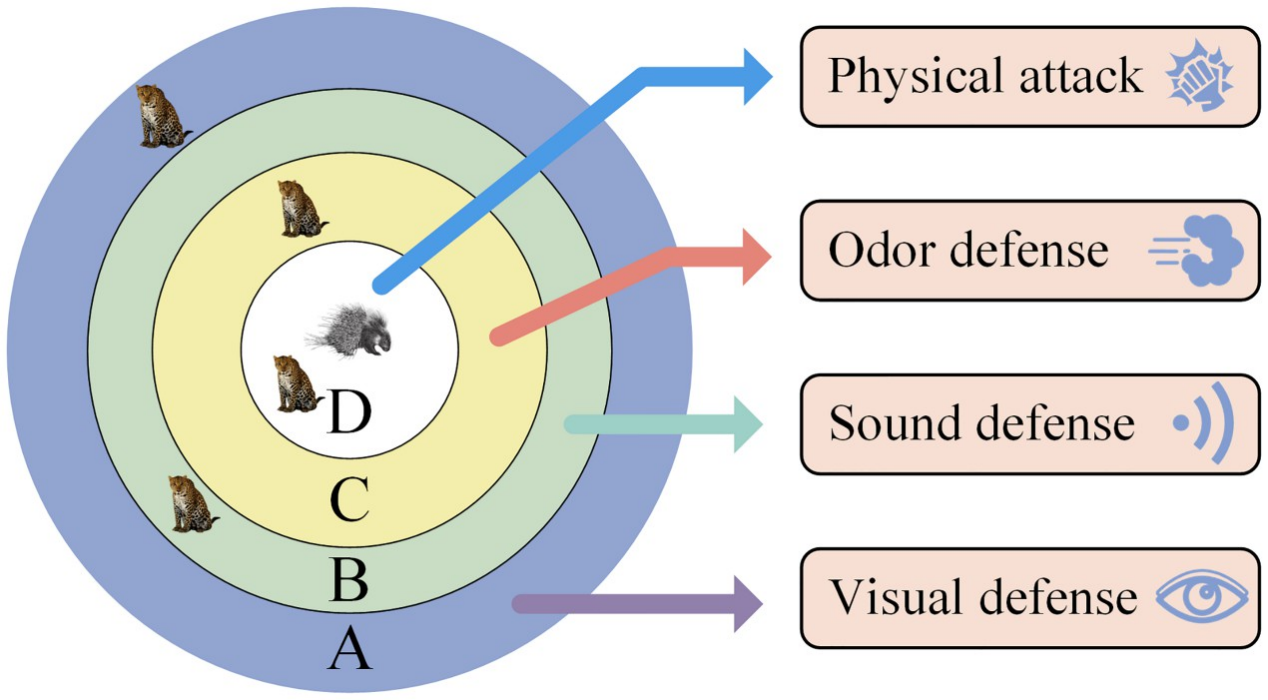
Crested porcupine defense area.

CPO can simulate various defensive behaviors of the crested porcupine, facing different degrees and types of threats and adopting the corresponding defensive behaviors. When the degree of threat gradually increases, the aggressiveness will also increase, and the search space is visualized as shown in Fig 7. The four regions from A to D represent four kinds of defense strategies, and the predator in region A is the farthest away from the porcupine, with the lowest aggressiveness of the defense strategy. The closer the region D is, the closer the predator is to the porcupine, and the aggressiveness of the defense strategy gradually increases. the predator in region D is the closest to the porcupine, and the aggressiveness of the defense strategy is the highest. The defense strategies represented from A to D are visual, sound, odor, and physical attacks, in that order. the first two defense strategies are known as the exploratory behaviors of CPOs, and the last two are known as the exploitative behaviors of CPOs [50].

Like other population-based metaheuristic algorithms, CPO first initializes a set of individuals to start the search process, as shown in Eq (9).

$$\begin{array}{c} {\vec{X}}_{i}=\vec{L}+\vec{r}\times (\vec{U}-\vec{L})|i=1,2\cdots ,N^{\prime } \end{array}$$ (9)

Where *N*′ denotes the number of individuals in the population size, ${\vec{X}}_{i}$ is the i-th candidate solution in the search space, $\vec{U}$ and $\vec{L}$ are the upper and lower bounds in the search range, respectively. $\vec{r}$ is a random number between 0 and 1.

In order to maintain population diversity while accelerating convergence, the CPO modeled a strategy where not all crown porcupines would turn on the defense mechanism, but only those that received a threat. This is the cyclic population reduction technique (CPR). In this strategy, some crested porcupines are first allowed to exit the population during the optimization process to speed up convergence, and then they are allowed to reenter the population to increase diversity and avoid falling into local extremes. The mathematical model is shown in Eq (10).

$$\begin{array}{c} N=N_{\text{min}}+(N^{\prime }-N_{\text{min}})\times (1-(\frac{t\%\frac{T_{\text{max}}}{T}}{\frac{T_{\text{max}}}{T}})) \end{array}$$ (10)

Where *T* is the variable that determines the number of cycles, *t* is the current number of function evaluations, *T*_max_ is the maximum number of times the function evaluates, % represent the remainder or modulus operator, *N*_min_ is the minimum number of individuals in a newly generated population [51].

The four defenses are described in detail below.

(1) Visual defense. When the crested porcupine notices a predator approaching, it quickly raises and flaps its quills in defense, with the goal of intimidating the predator, which is left with a choice between approaching and moving away. The predator approaching shortens the distance between them and speeds up area exploration, while staying away increases the distance between them and helps to explore distant areas. The mathematical model equation for this behavior is shown in Eq (11).
  $$\begin{array}{c} \vec{x_{i}^{t+1}}=\vec{x_{i}^{t}}+\tau_{1}\times |2\times \tau_{2}\times \vec{x_{CP}^{t}}-\vec{y_{i}^{t}}| \end{array}$$ (11)
  Where $\vec{x_{CP}^{t}}$ is the optimal solution of the function evaluating t, $\vec{y_{i}^{t}}$ is a vector generated between the current crested porcupine and a randomly selected crested porcupine from the population, representing the position of the predator at iteration t. *τ*_1_ is a random number based on a normal distribution, *τ*_2_ is a random value in the interval [0, 1] [52].
(2) Sound defense. When a predator approaches a crested porcupine, the crested porcupine will mimic sounds to send a threat to the predator. The predator hears the sound and chooses to continue to approach, stand still, or move away. The mathematical model equation for this behavior is shown in Eq (12).
  $$\begin{array}{c} \vec{x_{i}^{t+1}}=(1-\vec{U_{1}})\times \vec{x_{i}^{t}}+\vec{U_{1}}\times (\vec{y}+\tau_{3}\times (\vec{x_{r1}^{t}}-\vec{x_{r2}^{t}})) \end{array}$$ (12)
  Where r1 and r2 is two random integers between [1,N], *τ*_3_ is a random value generated between 0 and 1. $\vec{y}$ represent predator positions [52].
(3) Odor defense. Crested porcupines secrete a fishy odor, and the diffusion of the odor prevents the gradual approach of predators. The mathematical model equation for this behavior is shown in Eq (13).
  $$\begin{array}{c} \begin{array}{cc} \vec{x_{i}^{t+1}} & =(1-\vec{U_{1}})\times \vec{x_{i}^{t}}+\vec{U_{1}}\times (\vec{x_{r1}^{t}}+S_{i}^{t}\times (\vec{x_{r2}^{t}}-\vec{x_{r3}^{t}})\\ & -\tau_{3}\times \vec{\delta }\times \gamma_{t}\times S_{i}^{t}) \end{array} \end{array}$$ (13)
  Where r3 is a random number between [1,N], *δ* is a parameter used to control the search direction, *γ*_*t*_ is a defense factor, *τ*_3_ is a random value between 0 and 1, $S_{i}^{t}$ is the odor diffusion factor, which is used to optimize the odor diffusivity of the control process [52].
(4) Physical attack. When a predator enters the neighboring area of a crested porcupine and the distance between them reaches its closest point, the crested porcupine will use its quills as a weapon to physically attack. The mathematical model equation for this behavior is shown in Eq (14).
  $$\begin{array}{c} \vec{x_{i}^{t+1}}=\vec{x_{CP}^{t}}+(\alpha (1-\tau_{4})+\tau_{4})\times (\delta \times \vec{x_{CP}^{t}}-\vec{x_{i}^{t}})-\tau_{5}\times \delta \times \gamma_{t}\times \vec{F_{i}^{t}} \end{array}$$ (14)
  Where $\vec{x_{CP}^{t}}$ is the optimal solution obtained, representing the crested porcupine, $\vec{x_{i}^{t}}$ is the position of the i-th individual at iteration t, representing the predator at that position, *α* is the convergence speed factor, *τ*_4_ is a random value in the interval [0, 1] [52].

### CPO-CNN model building

Considering the superiority of CNN in processing image data, the complexity of PQDs, and the inconvenience of human experience in feature selection, this paper uses CNN structure for feature extraction, feature selection and classification of time-frequency images containing complex features. At the same time, the CPO optimization algorithm is used to optimize the hyperparameters in the CNN structure to construct a neural network model with better generalization performance and classification accuracy, so as to realize the accurate classification of complex disturbances in power quality.

In the constructed classification prediction model, different layers undertake different functions and tasks respectively [53]. The input layer accepts the time-frequency converted image data, and the convolutional layer performs convolutional operations through multiple convolutional kernels to extract the perturbed local features. The activation function uses the ReLU function to convert linear transformations into nonlinear transformations, enabling the model to learn complex patterns and features. The pooling layer reduces the size of the feature map by maximum pooling operation to reduce the feature dimensions and retain important features. The batch normalization layer normalizes the data to stabilize the training process and accelerate convergence. The fully connected layer is trained with weights to select the most important perturbed features extracted to optimize the classification effect. The output layer uses softmax function to generate the final classification results.

In order to select the CNN network structure with the best classification effect, the training and classification accuracy of neural networks with different network structures are compared with the same parameters such as the maximum number of training times, the number of neurons per layer, and the batch sample size, and the optimal model structure is finally determined. The first is the input layer, which determines the input size of the spectrogram, with an image size of 256 × 256 × 1. The second, third and fourth layers are all convolution blocks containing a convolutional layer with a pooling layer, and the input data is convolved by their translation to extract feature information. In this example, the second layer convolution kernel size is set to 5 × 5 with a step size of 1, the third layer convolution kernel size is set to 3 × 3 with a step size of 1, and the fourth layer convolution kernel size is set to 3 × 3 with a step size of 1. Pooling is maximal pooling, and the pooling window size is 4 × 4 with a step size of 1. The activation function is the ReLU function. the one-dimensional array obtained from the flattened feature extraction by the CNN network is the output. The number of neurons in the fully connected layer is 10, which matches the number of model classification categories. The softmax function is used in the output layer to produce the final classification accuracy and the loss function is also calculated.

**Fig 8:**
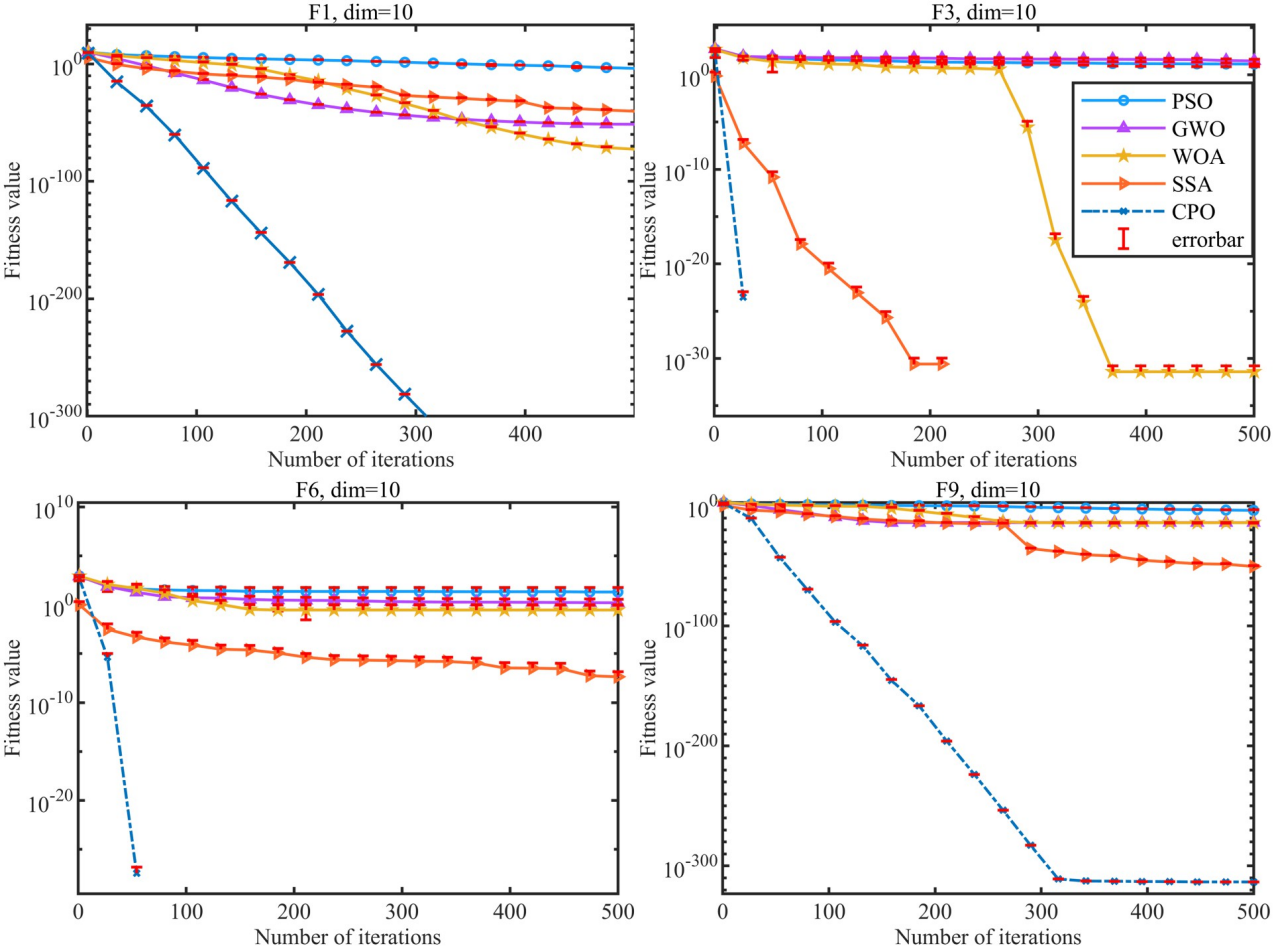
Test function evolution curve.

The hyperparameters of the neural network model are usually manually adjusted step by step through manual experience, which cannot ensure good results while paying a lot of time cost, and overfitting and underfitting occur frequently. While determining the structure of the CNN network, the CPO algorithm is applied to optimally adjust the hyperparameters in the CNN such as the learning rate, batch sample size, regularization coefficient, etc. The population size of the CPO is set to 10, and the maximum number of iterations is set to 20. The CPO algorithm, which combines the characteristics of global search and local optimization, shows excellent robustness and high efficiency in solving complex problems. The optimal solutions of single-peak function (F1), basis function (F3), hybrid function (F6) and combined function (F9) are solved in dimension 10 by using the crown porcupine optimization algorithm (CPO), particle swarm optimization algorithm (PSO), grey wolf optimization algorithm (GWO), whale optimization algorithm (WOA), and sparrow optimization algorithm (SSA), respectively, and analyzed and compared, and their convergence curves are shown in Fig 8.

Compared with other optimization algorithms, Fig 8 fully demonstrates that the CPO algorithm has a stable convergence curve and faster convergence speed, and possesses better performance compared with other algorithms, which effectively guides the optimization process in the direction of the optimal solution. The effectiveness of the CPO algorithm in dealing with a variety of complex real-world optimization problems has been verified. Also to avoid the follow-on effects, the algorithm was run 10 times for each case separately and the standard deviation error bars were used to indicate the stability of the results. From the figure it can be seen that the error of the algorithm for each run is within a small range and the results are stable. Therefore, the CPO algorithm can be practically applied to the neural network model to find the optimal combination of hyperparameters through continuous iteration to improve the performance of the model in practical applications, so as to better classify the complex disturbances of power quality of the grid, and then improve the security and stability of the power grid.

The setting and adjustment of the parameters of the CPO algorithm itself also determine the classification performance of the model. In the CPO optimization algorithm, the population size, maximum number of iterations, dimension parameter, boundary parameter, and objective function are adjustable parameters. The population size affects the breadth and diversity of the search, and a larger population size can provide a more comprehensive search, but will make the computational cost of training the CNN higher. The maximum number of iterations determines the convergence speed of the algorithm and the adequacy of the search, too low may result in failure to find the optimal solution. The dimension parameters are consistent with the hyperparameters to be optimized. The boundary parameters include the lower and upper bounds of the optimization parameters, which determine the search range and may limit the model performance if not set properly. The objective function, on the other hand, is used to evaluate the effectiveness of the optimization and directly affects the effectiveness of the optimization process. Through the adjustment of these parameters, the optimal hyperparameters during CNN training are ensured, and the training efficiency, complexity and classification performance of the model are controlled. In this paper, after going through many adjustments of the parameters, it is determined that the population size of CPO is set to 10, the maximum number of iterations is set to 20, the dimensionality parameter is 3, the lower and upper bounds of the batch sample size are 32 and 128, the lower and upper bounds of the regularization coefficients are 10^−4^ and 10^−2^, the lower and upper bounds of the learning rate are 10^−4^ and 10^−1^, and the objective function is the accuracy of the model. The classification performance of the classification model can be optimized using the adjusted CPO parameters.

**Fig 9:**
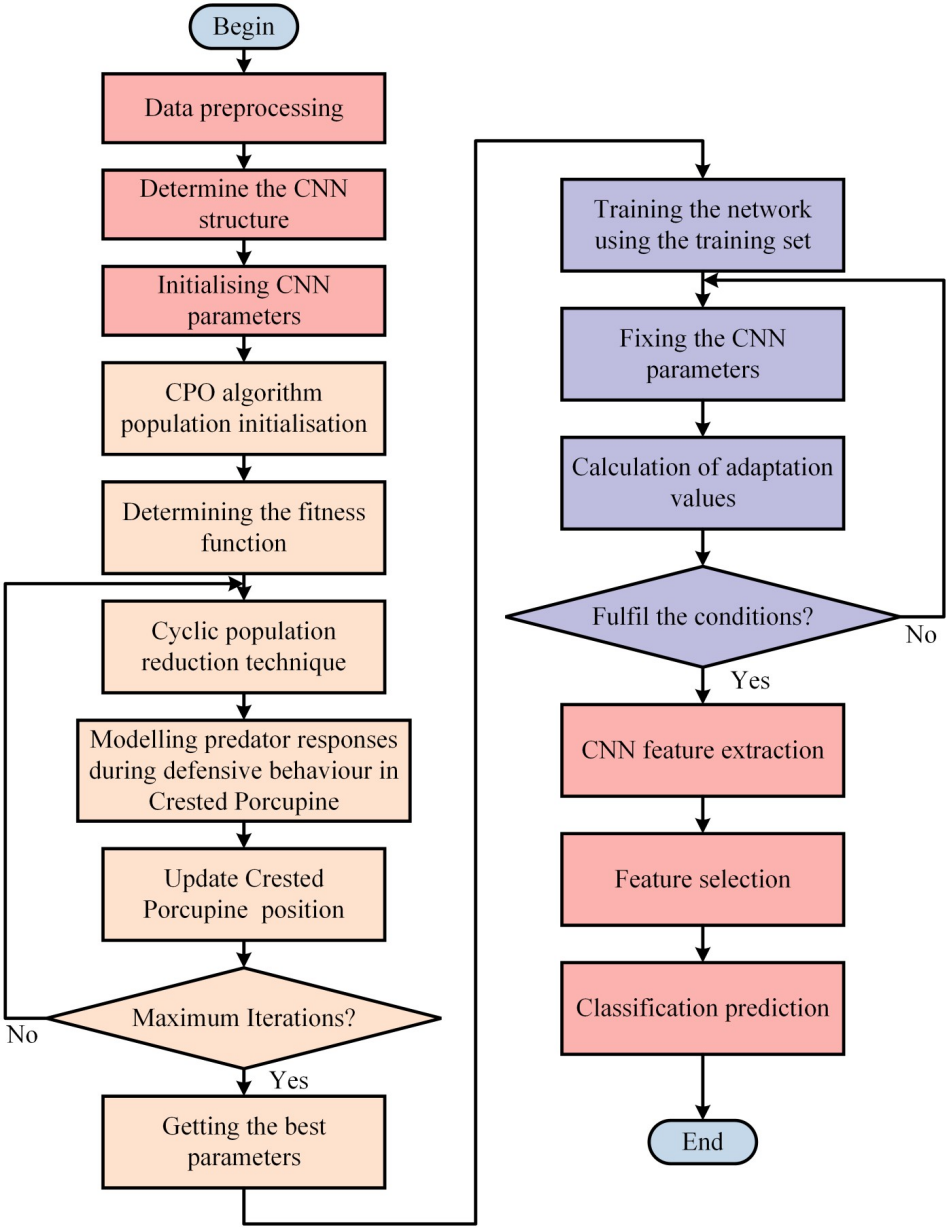
CPO-CNN model flowchart.

In summary, this paper proposes a CPO-CNN classification prediction model, whose flowchart is shown in Fig 9 and is implemented as follows.

Step 1. The time-frequency image after time-frequency conversion of the original signal is preprocessed, and the training set and test set are divided in order to construct a dataset as the input of the model.
Step 2. Combine the advantages of CNN in image processing and feature extraction, capture the hidden relationship of power quality disturbance data, and determine the CNN structure after experimental comparison to construct the optimal CPO-CNN model.
Step 3. Initialize the CNN network parameters and use the CPO optimization algorithm to adjust the hyperparameters of the model.
Step 4. Each crested porcupine adopts different defense strategies according to the predator position and updates its own position according to the formula, then detects its own boundaries, stops iterating when it reaches a certain fitness value or the maximum number of iterations, and outputs the optimal hyperparameters.
Step 5. Train the neural network, substitute the generated optimal hyperparameters into the model and determine whether the optimal adaptation is reached.
Step 6. Perform feature extraction, feature selection and classification prediction on the input data to obtain the final prediction results.

## Experimental simulation and analysis

In this paper, experimental simulations are performed in MATLAB R2022a version. Waveform signals containing different PQDs are generated by program simulation according to international standards and these raw signals are used as experimental objects. Time-frequency features are extracted from the original signals by fast S-transform to form a data set. After dividing the training set and test set these disturbance signals are classified using CPO optimized CNN model. Finally, the classification results are analyzed and compared with those of other traditional classification models to verify the accuracy and effectiveness of the proposed model classification method.

### Generate disturbance datasets

The offshore wind power integration into the power grid is taken as the research background, and the PQDs on the AC bus of the power grid are taken as the research object. According to the IEEE 1159-2019 standard, 10 kinds of PQDs mathematical models are established, including 6 kinds of single disturbances and 4 kinds of double complex disturbances. The bus voltage level is 220KV, the fundamental frequency is set to 50HZ, the sampling frequency is set to 6.4kHZ, and the sampling duration is 10 fundamental waveform cycles, totaling 1280 sampling points. Under the above conditions, MATLAB is used as a tool to randomly simulate the generation of 10 power quality disturbances, 500 samples for each disturbance signal, totaling 5000 samples. The time of occurrence of the disturbance signals are all randomized, and divided into training set and test set according to the ratio of 7:3 for the training and testing of the model. Finally, the generated individual time series signals are batch processed by writing a fast S-transform program, and the resulting 2D images are saved as the required input data set for the model. In order to simplify the simulation, the disturbance waveforms in this paper are all single-phase. 10 types of disturbance signal categories and classification labels are shown in Table 2.

**Table 2:** Disturbance categories and label definitions.

Disturbance signal category	Label	Disturbance signal category	Label
Swell	C1	Oscillatory transient	C6
Sag	C2	Harmonic with swell	C7
Interruption	C3	Harmonic with sag	C8
Harmonic	C4	Harmonic with interruption	C9
Flicker	C5	Flicker with harmonics	C10

### Hyperparameter optimization results

Model accuracy is affected by hyperparameters such as learning rate, regularization factor, and batch size. The batch size determines the number of samples to be trained in each iteration, the learning rate determines how long it takes for the network to obtain the global optimal solution, which in turn ensures the training efficiency and network generalization, and the regularization factor is used to prevent the occurrence of overfitting. The proposed CPO algorithm and the PSO algorithm and WOA algorithm, which are involved in the subsequent comparisons, are used to optimize the above hyperparameters, and the objective function of all three optimization methods is the accuracy of the trained network on the training set. The classification effect is improved by optimizing the hyperparameters, and the hyperparameter optimization results are shown in Table 3.

**Table 3:** Hyperparameter optimization results.

Parameterization	Limit	PSO	WOA	CPO
L2 regularization	[10^−4^,10^−2^]	0.0015	0.0092	0.0026
Learning rate	[10^−4^,10^−1^]	0.0048	0.0040	0.0012
Batch sample size	[32, 128]	73	105	84

### Experimental results and comparative analysis

**Fig 10:**
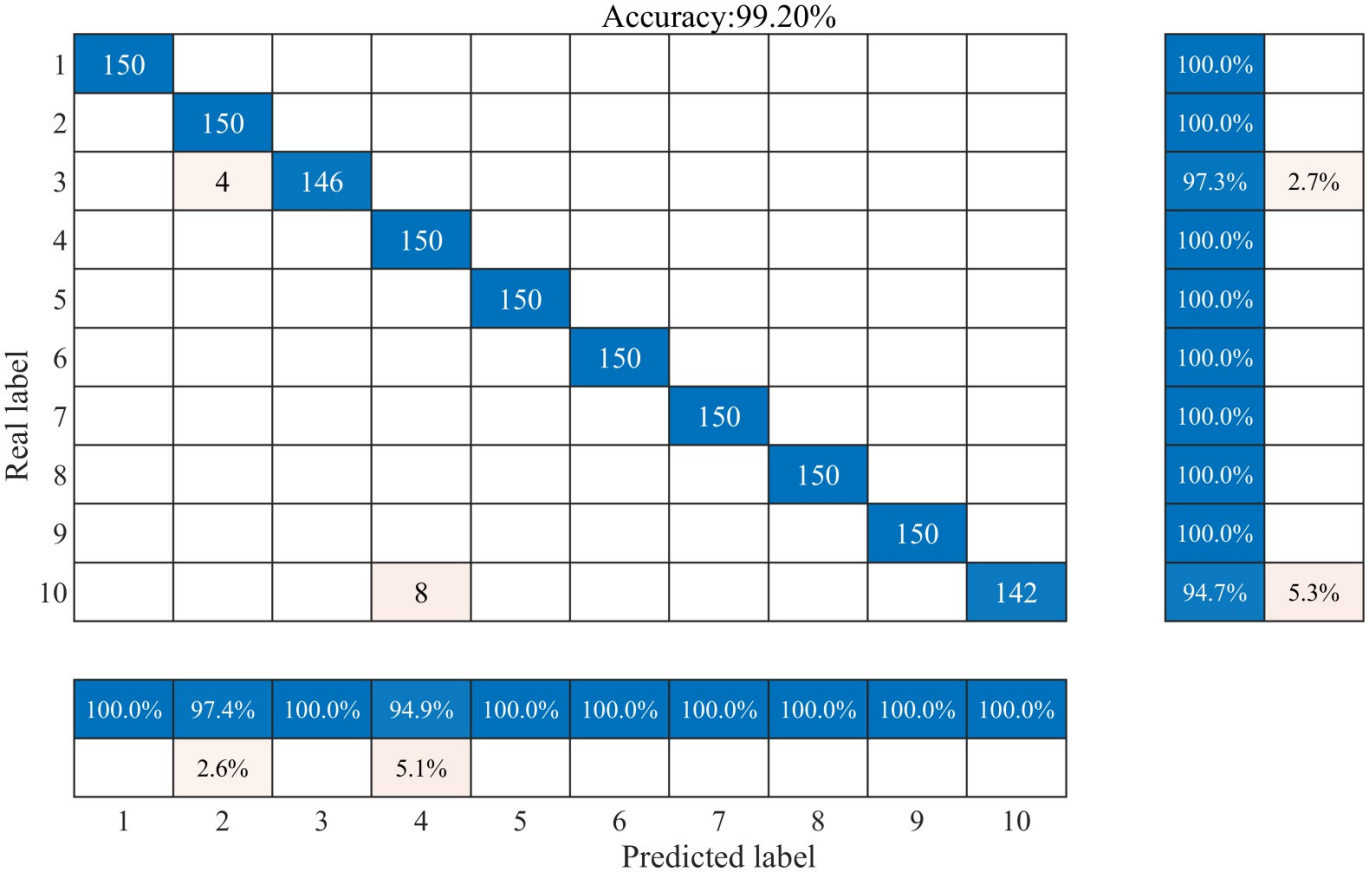
CPO-CNN model classification prediction results.

**Fig 11:**
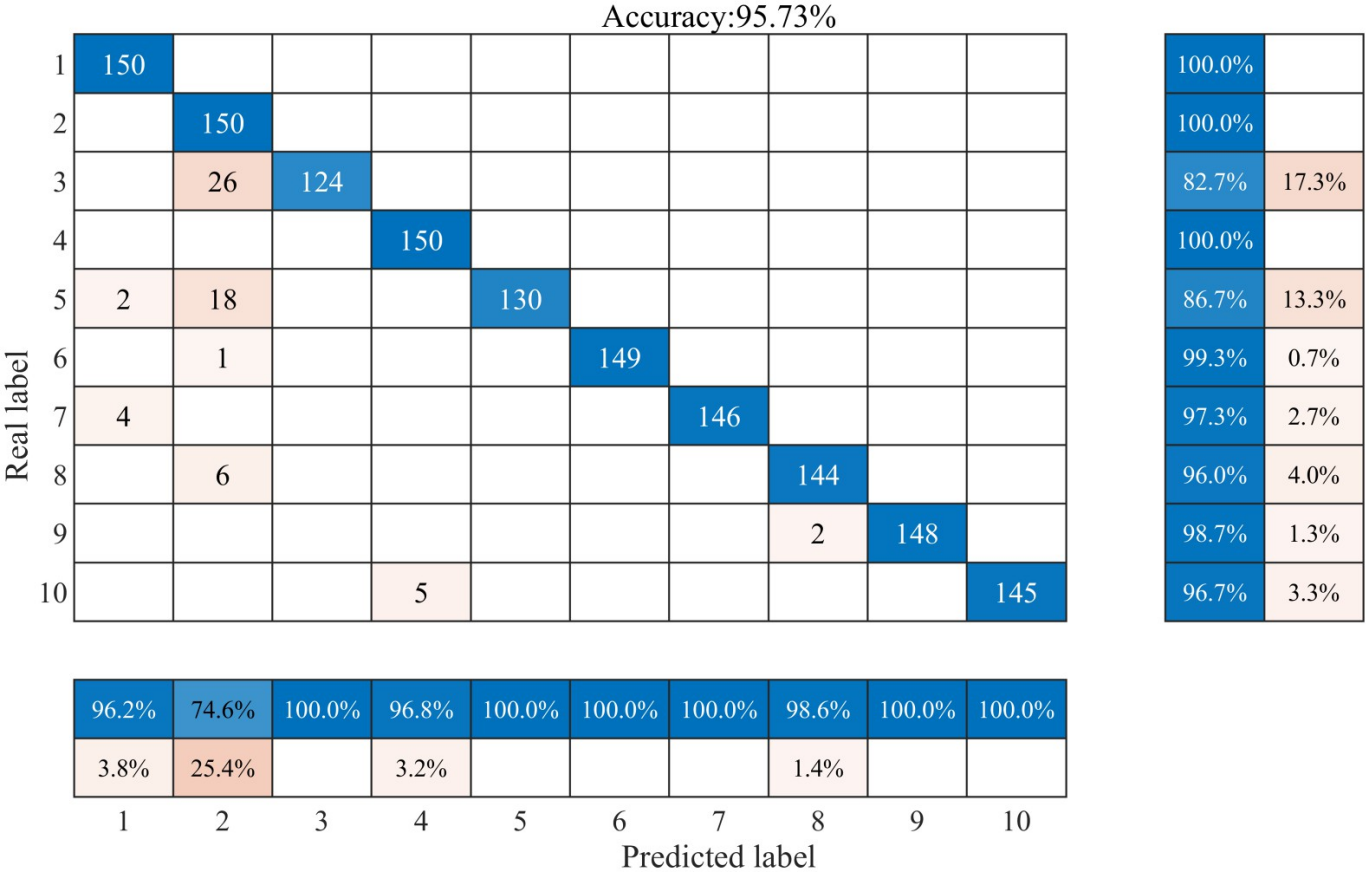
CNN model classification prediction results.

**Fig 12:**
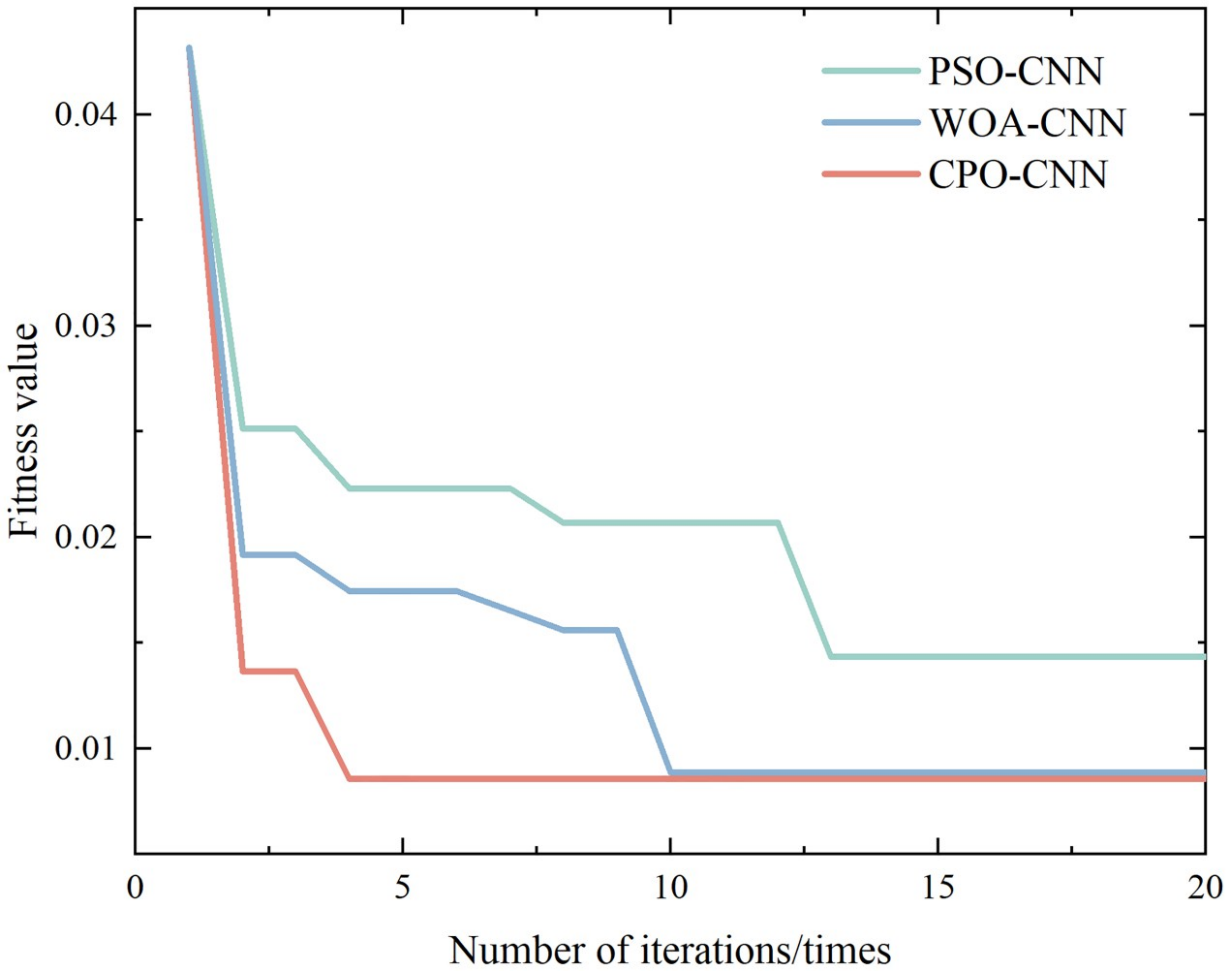
Fitness curves for each algorithm.

The first 350 sets of data for each sample type are extracted to form the training set, and the remaining 150 sets of data form the test set. In order to better verify the effect of the CPO algorithm on the model performance and the specific classification performance of the proposed model in this paper, the training prediction was repeated 100 times for the CNN model without parameter adjustment and the CPO-CNN model with parameters adjusted to the optimum, respectively, and the proposed model did not suffer from the phenomenon of overfitting at the end of model training. The classification results and confusion matrix of the CPO-CNN model for 10 different interference signals are shown in Fig 10, and the corresponding fitness curves are shown in Fig 12. The classification prediction results and confusion matrix of the CNN model are shown in Fig 11.

As can be seen from Fig 10, the CPO-CNN model is more sensitive to changes in the actual data, and the overall classification accuracy reaches 99.2%, with high accuracy for each individual disturbance. The horizontal coordinates in the figure are the real labels and the vertical coordinates are the predicted labels, the blue region on the diagonal represents the classification accuracy region for each category of disturbance, and the numbers represent the number of disturbance groups that are recognized accurately, 150 groups proves that all of them are recognized accurately. The light pink region is the region of misclassification. The table on the right side of the confusion matrix represents the proportion of samples predicted to belong to a particular category that actually belong to each category, which helps to understand the accuracy of the model’s predictions on each category. The lower table represents the proportion of samples that actually belonged to a category that were predicted to belong to a category, which helps to understand the distribution of each actual category across the predicted categories. From the distribution of values in the confusion matrix and the tables on both sides, it can be seen that the proposed model is less effective in categorizing the labels 3 and 10. The misidentification of voltage interruptions as voltage sags is due to the fact that both types of labels are characterized by a decreasing change in amplitude, which is misidentified by the model. The misidentification of flicker and harmonic mixed disturbance signals as harmonic signals is due to the failure of the model to recognize flicker signals among harmonic signals when dealing with complex mixed disturbances. However, removing the above two types of labels, the proposed model is able to achieve complete identification, which further validates its effectiveness in classifying PQDs.

The comparison clearly shows that the CPO-CNN method proposed in this paper is 3.47% better than CNN in terms of classification accuracy, which indicates that the CPO-CNN method achieves more superior classification results in problem solving. From the distribution of the values of the confusion matrix with the table on both sides, it can be seen that the CNN model shows errors in both single disturbance and complex multiple disturbance classification, with the lowest accuracy for the disturbance labeled as 3. This indicates that the CNN model needs to be improved in capturing data features and classification prediction, and further optimization and improvement is needed to increase the classification accuracy.

In order to more comprehensively assess the effect and superiority of the CPO algorithm on the performance enhancement of the CNN classification model, as well as to improve the credibility and applicability of the model, multiple optimization algorithms are used to optimize the CNN classification prediction model, and the dataset is the same dataset. The proposed model in this paper is compared and analyzed with CNN model, CNN-SVM model, PSO algorithm optimization model and WOA algorithm optimization model. The hyperparameter optimization results are referred to Table 3.

Fig 12 shows the fitness curves of different classification algorithms in optimizing the CNN hyperparameters. the CPO algorithm has a more stable convergence curve and a faster convergence speed, and identifies the global optimal threshold of the CNN neural network after 4 iterations. the WOA algorithm follows closely, and achieves the global optimal threshold after about 10 iterations. the PSO algorithm has the worst effect, and is also more prone to fall into the local optimum in the course of iterations. The PSO algorithm is the least effective, and is more likely to fall into local optimization during the iteration. It also reflects the superiority of CPO-CNN model in recognizing and classifying complex disturbances.

**Fig 13:**
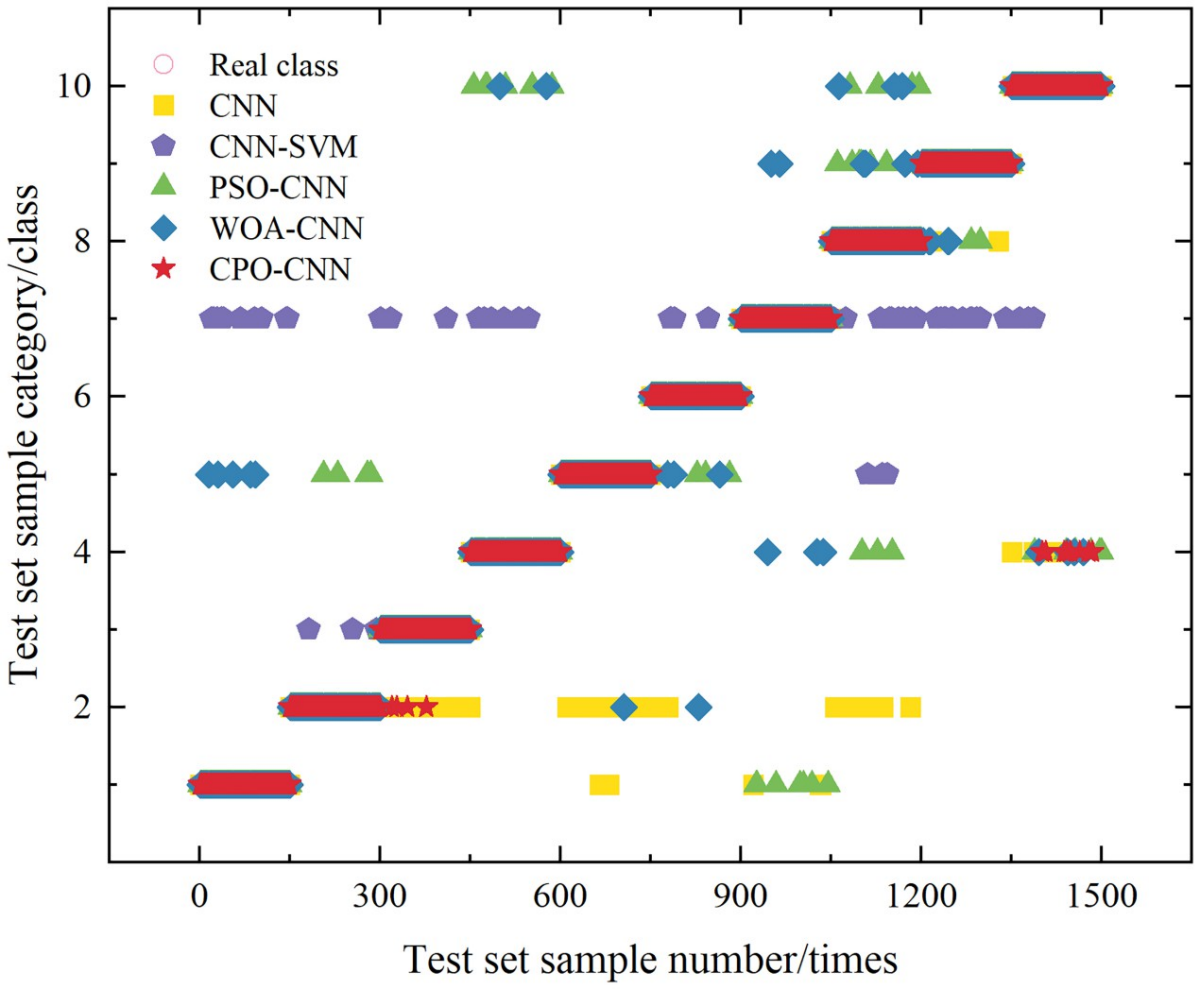
Comparison of classification results.

Fig 13 shows the comparison of classification effectiveness between the models. As can be seen from the figure, the CNN model that has not been optimized by the intelligent optimization algorithm has the worst classification ability, with more scattered points distributed in the coordinates, which represent the model’s misclassification when analyzing and processing these disturbances, and classifies disturbances belonging to this category into other categories. The CPO-CNN model has the highest classification accuracy, and the classification categories are basically the same as the real categories, with only a small number of errors in evaluating the complex multiple disturbances and voltage interruptions with fewer errors. In conclusion, the CPO-CNN classification model has superior classification performance.

In order to evaluate the performance of the methods more intuitively, the classification accuracy of the test set of each model is shown in Table 4, which shows that each method achieves high accuracy, but compared with the model proposed in this paper, there is still a certain gap in the recognition of some categories of disturbances, and it cannot completely and accurately recognize each category of disturbances. The CPO-CNN classification model achieves 100% classification accuracy and outperforms other classifiers in these disturbance categories, achieving excellent classification results, except for C3 and C10. There are individual misclassification cases in C3 and C10, with an accuracy of 97.3% for C3 and 94.7% for C10. The differences in the model’s learning ability and the features of the waveform samples lead to the differences in the accuracy and results of the two differences in labeling accuracy and misclassification of results. However, the CPO-CNN model still has the highest average accuracy of 99.2%, and the overall classification performance is still the strongest.

**Table 4:** Classification accuracy for each model test set.

Label	Category	Model classification accuracy				
		CNN	CNN-SVM	PSO-CNN	WOA-CNN	CPO-CNN
C1	Swell	100%	93.3%	100%	96.7%	100%
C2	Sag	100%	98%	97.3%	100%	100%
C3	Interruption	82.7%	98%	100%	100%	97.3%
C4	Harmonic	100%	96%	96%	98.7%	100%
C5	Flicker	86.7%	100%	100%	99.3%	100%
C6	Oscillatory transient	99.3%	97.3%	97.3%	97.3%	100%
C7	Harmonic with swell	97.3%	100%	95.3%	96.7%	100%
C8	Harmonic with sag	96%	92%	91.3%	95.3%	100%
C9	Harmonic with interruption	98.7%	92%	98.7%	96.7%	100%
C10	Harmonic with flicker	96.7%	98%	95.3%	97.3%	94.7%
Overall accuracy		95.73%	96.47%	97.13%	97.8%	99.2%

Table 5 shows the average CPU runtime and overall accuracy of each model by testing each method multiple times, giving a more comprehensive picture of the performance of each classifier. Although CNN has the shortest run time, it is the worst at classifying and predicting interference. As the model complexity increases, the average runtime gradually increases, and the increase in accuracy comes at the cost of longer runtimes. The model proposed in this study slightly reduces the runtime compared to WOA-CNN, but the accuracy is the highest among all models. This result indicates that CPO-CNN has the most superior overall performance.

**Table 5:** CPU runtime and accuracy for each model.

Classifier	Average running time	Overall accuracy
CNN	16.42s	95.73%
CNN-SVM	30.88s	96.47%
PSO-CNN	65.74s	97.13%
WOA-CNN	89.06s	97.8%
CPO-CNN	80.57s	99.2%

Comprehensive comparative analysis shows that the CNN model without parameter optimization has the worst classification effect, and the CPO-CNN model improves the classification accuracy by 3.47%, 2.73%, 2.07%, and 1.4% than CNN,CNN-SVM, PSO-CNN, and WOA-CNN models, respectively. It shows high prediction accuracy. This stable, accurate and effective classification method provides a reliable support for offshore wind grid-connected power quality disturbance classification.

## Conclusion

In this paper, for the problem of detecting and classifying power quality disturbances in the grid under the situation of large-scale offshore wind power grid-connection, we propose to use fast S-transform for time-frequency conversion, combined with CNN data-driven model and tuned with CPO algorithm to realize the classification of single and complex disturbances in power quality. The following conclusions are mainly obtained.

1. A fast S-transform time-frequency feature extraction model and a CPO-CNN classification prediction model are designed and established, and the original signal waveform generated by simulation is converted to time-frequency, and then the input data is used to drive the classification model for feature extraction, feature selection and classification analysis. The results show that the classification model fits appropriately and the classification accuracy reaches 99.2%, which can accurately classify the disturbances.
2. By comparing and analyzing the optimization results of different optimization algorithms with CPO algorithm in the optimization of four test functions, the convergence curve of CPO algorithm is characterized by stability and speed. This indicates that the CPO algorithm has good robustness and high efficiency in solving complex problems and provides an effective solution to the model parameter optimization problem.
3. The CNN model based on the CPO optimization algorithm improves the classification accuracy by 3.47% compared to the traditional CNN, and 2.73%, 2.07% and 1.4% compared to the CNN-SVM and other optimization algorithms optimizing the CNN model, respectively. This proves that the classification model under the CPO-based algorithm has higher classification accuracy and stability. It provides a new classification method for offshore wind power grid-connected power quality disturbance monitoring.

In the next step of the research, the problem of disturbance classification under noise and other disturbances will be further considered to improve the classification accuracy under different decibel noise. Although the model proposed in this paper already has a good classification effect, in a power grid integrated with offshore wind energy, multiple disturbances, including triple and more than triple disturbances, often occur at the same time, and better neural network structure and more advanced algorithms are still needed to further improve the model. Consideration also needs to be given to how to improve accuracy while reducing runtime. In order to meet the realistic needs, the system needs to be further optimized and improved.
